# *Nanomaterials* 2021 Best Paper Awards: Announcement and Interview with the Winner—Xin Huang

**DOI:** 10.3390/nano12030450

**Published:** 2022-01-28

**Authors:** Nanomaterials Editorial Office

**Affiliations:** MDPI AG, St. Alban-Anlage 66, 4052 Basel, Switzerland; nanomaterials@mdpi.com

After an extensive voting period, we are proud to present the Best Paper Award to “Nanomaterials for the Removal of Heavy Metals from Wastewater [1]” by the corresponding authors Dr. Baohong Hou and Dr. Xin Huang.


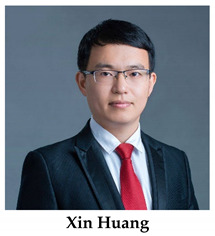


Xin Huang (Orcid 0000-0003-3358-5044) received his PhD in Chemical Engineering from Tianjin University in 2016. From 2016 to 2018, he worked as an associate research fellow at the Collaborative Innovation Center of Chemical Science and Engineering (Tianjin) under the supervision of academician Prof. Jingkang Wang. He was also a key member of the National Engineering Research Center of Industrial Crystallization Technology (NERCICT). Since the end of 2018, he has worked as an assistant professor at Tianjin University in the group of Prof. Hongxun Hao of the School of Chemical Engineering and Technology and NERCICT. During this period, he has also worked as a visiting scholar in the Department of Chemical Engineering, Imperial College London, with the cooperation supervisor Prof. Jerry Heng. His areas of interest are industrial crystallization, including polymorphism of drugs, functional crystal materials and nanomaterials. He has authored over 100 peer-reviewed papers (first author and corresponding author in more than 50 papers) with more than 1000 citations. Moreover, he has authored 12 patents. Now, he is the Guest Editor for *Current Pharmaceutical Design* (IF 3.116) and *Crystals* (IF 2.589). He has been funded as a principal investigator in many national, regional and enterprise cooperation projects.

On behalf of the *Nanomaterials* Editorial Office staff and Award Evaluation Committee, we congratulate Dr. Baohong Hou and Dr. Xin Huang on their excellent performance and wish them all the best for their future careers.

## Interview with the Winner

### 1. Could You Briefly Introduce Yourself to the Readers?

I am the corresponding author of the awarded paper “Nanomaterials for the Removal of Heavy Metals from Wastewater”. I am a teacher at school of Chemical Engineering and Technology, Tianjin University. Now I work in the research group of National Engineering Research Center of Industrial Crystallization Technology.

### 2. What Are You Currently Researching and Why Did You Choose this Research Field?

Now, my research interests focus on functional crystal materials and nanomaterials. As for the reason, maybe this is my own interest. I like the colorful and multifunctional crystals and I am also confidential with their great practical potential in control component and optical device.

### 3. Which Research Topics Do You Think Will Be of Particular Interest to the Research Community in the Coming Years?

This is a huge question. It’s difficult to talk about other research fields. But, in the crystallization and nanomaterials research area, I think the preparation and production of highly efficient materials that handling heavy metals and organic contaminant in actual wastewater is meaningful. A series of materials have been developed these years to deal with water contamination. But their effect, efficient as well as the economy problems are always existing. So, if we can solve these problems, I think the research community will be inspired.

### 4. As a Young Researcher, what Was the Biggest Challenge You Encountered in Your Research Journey? How Did You Solve it?

The balance between teaching and research work, the intensified competition during the promotion are both challenges for me. Thanks to the help and support from our excellent team, I’m feeling pretty good now.

### 5. Can You Briefly Describe the Key to a Happy Laboratory Life?

First of all, the most important is your desire and love of scientific research. You may enjoy a surprising finding or an interesting phenomenon. Of course, a nice team that you work with is also essential. 

### 6. If You Have the Opportunity, Will You Actively Apply to Attend Academic Conferences? What Do You Think You Can Learn from Participating in Conferences That Is Different from Working in a Lab?

Sure, of course. During the past years, we often attend academic conferences and gave some oral presentations, both in China and other countries. But, due to the COVID-19, meetings are usually hold online during these two years. Anyway, these conferences are good opportunities for us, we can share our research progress with other researchers and discuss with them. In this process, we can obtain the latest research achievements from different research directions, and we may gain some scientific inspirations. This is an efficient method to get useful information.

### 7. Which Qualities Do You Think Young Researchers Need?

There are many required qualities for young researchers, like patience, creativity, passion, imagination. Of course, a good physical constitution is also important.

### 8. As an Open Access Journal. How Do You Think Open Access Impacts the Authors?

For the author, open access journals can help to increase the visibility and impact of their research findings. Open access journals can also provide a wider global readership than any subscription-based journal. Of course, this is helpful to increase the international influence of authors.

## References

[1] Yang J., Hou B., Wang J., Tian B., Bi J., Wang N., Li X., Huang X. (2019). Nanomaterials for the Removal of Heavy Metals from Wastewater. Nanomaterials.

